# Lysosomal Storage Disorders and Malignancy

**DOI:** 10.3390/diseases5010008

**Published:** 2017-02-27

**Authors:** Gregory M. Pastores, Derralynn A. Hughes

**Affiliations:** 1 Department of Medicine (Genetics), University College Dublin, Mater Misericordiae University Hospital, Dublin, Ireland; 2 Royal Free London NHS Foundation Trust, University College London, London NW3 2QG, UK; rmgvdah@ucl.ac.uk

**Keywords:** lysosome, gammopathy, multiple myeloma

## Abstract

Lysosomal storage disorders (LSDs) are infrequent to rare conditions caused by mutations that lead to a disruption in the usual sequential degradation of macromolecules or their transit within the cell. Gaucher disease (GD), a lipidosis, is among the most common LSD, with an estimated incidence of 1 in 40,000 among the Caucasian, non-Jewish population. Studies have indicated an increased frequency of polyclonal and monoclonal gammopathy among patients with GD. It has been shown that two major sphingolipids that accumulate in GD, namely, β-glucosylceramide 22:0 (βGL1-22) and glucosylsphingosine (LGL1), can be recognized by a distinct subset of CD1d-restricted human and murine type II natural killer T (NKT) cells. Investigations undertaken in an affected mouse model revealed βGL1-22- and LGL1-specific NKT cells were present and constitutively promoted the expression of a T-follicular helper (TFH) phenotype; injection of these lipids led to downstream induction of germinal center B cells, hypergammaglobulinemia, and the production of antilipid antibodies. Subsequent studies have found clonal immunoglobulin in 33% of sporadic human monoclonal gammopathies is also specific for the lysolipids LGL1 and lysophosphatidylcholine (LPC). Furthermore, substrate reduction ameliorated GD-associated gammopathy in mice. It had been hypothesized that chronic antigenic stimulation by the abnormal lipid storage and associated immune dysregulation may be the underlying mechanism for the increased incidence of monoclonal and polyclonal gammopathies, as well as an increased incidence of multiple myeloma in patients with GD. Current observations support this proposition and illustrate the value of investigations into rare diseases, which as ‘experiments of nature’ may provide insights into conditions found in the general population that continue to remain incompletely understood.

## 1. Introduction

Lysosomal storage disorders (LSDs) represent a heterogeneous group of inherited errors of metabolism (IEM), which result primarily from a disruption in the usual sequential degradation of macromolecules or their transit within the cell. As a consequence, there is progressive accumulation of incompletely processed cellular material within various tissue types, and corresponding disease-specific clinical manifestations. Individual disorders are infrequent to rare conditions.

Recent studies have revealed expanding roles for lysosomes, beyond substrate degradation, including involvement in energy homeostasis, generation of building blocks for cell growth, mitogenic signaling, the priming of tissues for angiogenesis and metastasis formation, and activation of transcriptional programs [1].

The occurrence of malignancy among patient with LSDs has not been systematically examined, except among a group of patients with Gaucher disease type 1 (GD1) in whom there is a recognized increase in the incidence of gammopathy and risk of developing multiple myeloma and possibly other hematological malignancies [2,3].

GD1 is an autosomal recessive IEM, caused by a deficiency of the lysosomal hydrolase acid β-glucosidase and the resultant accumulation of its primary substrate, glucosylceramide (GC), which in the systemic circulation is derived primarily from the turnover of senescent blood cell membranes [4]. Cardinal manifestations include the infiltration of bone marrow, liver, spleen, and lung by lipid-engorged macrophages (Gaucher cells). Acute and subacute forms of Gaucher disease, designated type 2 and 3 GD, respectively, manifest with primary central nervous system (CNS) involvement expressed as spasticity, oculomotor apraxia, and seizures with onset in infancy or early childhood, and leading to premature death. Although the designation GD1, by convention excluded patients with primary CNS involvement, investigations in the last decade have revealed a subset of adult patients and carriers have an increased risk of Parkinsonism/Parkinson disease (PD) [5]. The basis for the increased risk of PD remains to be more fully elucidated; studies have suggested GC accumulation promotes the aggregation of α-synuclein, aggregates of which are a constituent of Lewy bodies (a major pathologic hallmark of PD).

## 2. Gaucher Disease and Malignancy

Gammopathy is recognized as a comorbidity among adults with GD1, although formal assessment of its prevalence and the association with increased risk of monoclonal gammopathy and multiple myeloma had not been undertaken until recently.

Goldfarb and colleagues (1950) were the first to report on their findings of polyclonal (diffuse) hypergammaglobulinemia and Gaucher disease in a group of patients under the age of 30 [6]. Subsequent reports described the case of a patient with GD and monoclonal gammopathy, and another patient with GD and multiple myeloma [7,8]. During this time, there was uncertainty regarding a causal link, as concomitant osteolytic lesions and pathologic fractures may occur in patients with GD with or without myeloma. Bone disease, including lytic and sclerotic lesions, is a major GD-related complication, particularly among splenectomized individuals in the period prior to the introduction of enzyme replacement therapy (ERT). [Prior to the availability of ERT, splenectomy was undertaken to deal with bleeding complications from low platelet counts secondary to hypersplenism and in patients in whom a massively enlarged spleen resulted in great discomfort and/or mechanical problems for affected patients.] A further confounding factor was the realization that certain disorders associated with a high turnover of cellular membranes resulted in the generation of pseudo-Gaucher cells. [Gaucher-like or pseudo-Gaucher cells can be seen in acute lymphoblastic leukemia, multiple myeloma, myelodysplasia, Hodgkin’s disease, thalassemia, and disseminated mycobacterial infection [9]. Immunohistochemical studies show that Gaucher cells react for monocytic antibodies but differ from normal monocytes by a very strong expression of HLA-DR antigens, an active role in the chronic stimulation of the immune system [10]].

Rosenbloom and coworkers (2005) examined the incidence of cancers in patients with GD enrolled in the International Gaucher Registry (ICGG), an observational database that had been set up by Genzyme (now a Sanofi company) following the regulatory approval of alglucerase. [Alglucerase, which was purified from human placenta, was the first ERT for GD1, introduced in 1991.] Data on 2742 patients from the Registry was analyzed. When the database was locked to establish an analytic set, the majority of enrolled patients were young or middle-aged adults at the time of the last follow-up, and only 14% were older than age 60 [2]. Ten patients were reported to have multiple myeloma, yielding an estimated relative risk of 5.9 (95% confidence interval [95% CI]: 2.8, 10.8). The relative risk of cancer overall was 0.79 (95% CI: 0.67, 0.94), and the subgroups for cancers of the breast, prostate, colon and rectum, lung, and hematologic malignancies other than myeloma did not yield statistically significant higher risks. It was concluded that, in general, patients with GD are not at a highly increased risk of cancer, at least during early and middle age. However, there appeared to be a significantly higher risk of multiple myeloma.

De Fost and co-investigators (2006) examined the incidence and mortality of cancer in a total of 131 GD patients of mixed ancestry in a population from Western Europe, i.e., two Gaucher referral centers in Germany (Düsseldorf) and the Netherlands (Amsterdam). Fourteen GD patients of non-Ashkenazi-Jewish descent were identified; of these, five had hematologic malignancies [3]. These numbers correspond to an increased risk of cancer of 2.5 (95% CI 1.1–4.7) and an increased risk of hematologic cancer of 12.7 (95% CI 2.6–37.0) among GD patients compared to the general Dutch population. In particular, the incidence of multiple myeloma and hepatocellular carcinoma in the absence of pre-existing cirrhosis were highly elevated, with standardized rate ratios of 51.1 (95% CI 6.2–184) and 141.3 (95% CI 17.1–510.5), respectively.

## 3. Gaucher Disease and Hematologic Cancer

Allen et al. reported enhanced release of cytokines (specifically IL-6 and IL-10) from pathological GD macrophages, providing a putative pathological link between GD and associated lympho-proliferative disorders [11]. In a review of extant literature, Cotello and colleagues (2006) posited pathologic macrophages in GD may trigger B-lymphocyte polycolonal immunoglobulin secretion (directly via IL-1/IL-6 secretion), which could be reversed with the disappearance of antigenic stimulation, following the introduction of therapy [12]. On the other hand, multiple myeloma, which involved both clonal selection and mutations would not be reversed [12]. [Long-term antigenic stimulation may, in principle, also promote genomic instability in myeloma by engaging cytidine deaminases [13].] These hypotheses were in line with earlier observations by Brautbar and coworkers (2004), noting an incidence of polyclonal gammopathies that ranged between 14% and 25% among treated and untreated GD patients in the Israeli cohort based at Shaare Zedek Medical Center in Jerusalem [14]. Furthermore, there was a statistically significant percentage decrease per year of enzyme therapy in those with polyclonal but not monoclonal (1% of all patients) gammopathies [14]. Back then, the specific mechanistic link(s) mediating these observations had not been fully elucidated.

A new era was ushered in by studies undertaken in GD mouse models. Until recently, there had been no animal model of GD that could be sustained and available for appropriate investigations. The first knock-out mouse model generated by recombinant genetic techniques resulted in death shortly after birth, attributed to several factors including irregular respiration and poor feeding consistent with nervous system dysfunction, and alterations in the skin which resulted in increased permeability [15].

In 2010, a mouse model was generated with a conditionally deleted *GBA1* gene in hematopoietic and mesenchymal cell lineages. Analysis involving cytokine measurements, microarray analysis, and cellular immunophenotyping together revealed widespread dysfunction not only of macrophages, but also of thymic T cells, dendritic cells, and osteoblasts [16]. Additionally, the proliferation of GCase-deficient hematopoietic stem cells was inhibited significantly by both glucosylceramide and glucosylsphingosine (two major sphingolipids which accumulate in GD), suggesting that the “supply” of early thymic progenitors from bone marrow may be reduced in GBA deficiency. Subsequent studies revealed a plethora of immune cell aberrations, including alterations reminiscent of impaired T-cell maturation, aberrant B-cell recruitment, enhanced antigen presentation, and impaired egress of mature thymocytes [17]. In contrast to the profound defects in the thymus, there were only limited cellular defects in peripheral lymphoid organs, mainly restricted to mice with severe disease. The cellular changes in GCase deficiency were accompanied by elevated T-helper (Th)1 and Th2 cytokines that tracked with disease severity.

As chronic inflammation including B-cell activation is commonly observed in both inherited and acquired disorders of lipid metabolism, there was interest in studying putative cellular mechanisms underlying B-cell activation in Gaucher disease. Investigations along these lines revealed that β-glucosylceramide 22:0 (βGL1-22) and glucosylsphingosine (LGL1) can be recognized by a distinct subset of CD1d-restricted human and murine type II natural killer T (NKT) cells [18]. These studies showed human βGL1-22- and LGL1-reactive CD1d tetramer-positive T cells have a distinct T-cell receptor usage and genomic and cytokine profiles compared with the classical type I NKT cells. In contrast to type I NKT cells, βGL1-22- and LGL1-specific NKT cells constitutively expressed a T-follicular helper (TFH) phenotype. Furthermore, injection of these lipids led to an increase in respective lipid-specific type II NKT cells in vivo and the downstream induction of germinal center B cells, hypergammaglobulinemia, and production of antilipid antibodies. More recently, it has been shown that the clonal immunoglobulin found in 33% of patients with sporadic monoclonal gammopathies is also specific for the lysolipids LGL1 and lysophosphatidylcholine (LPC) [19]. The basis of this reactivity is not known, but suggests there may be an unrecognized disruption of lipid metabolism (whether intrinsic or extrinsic, e.g., diet-induced), resulting in the generation of an antigen that acts as a trigger and potential contributory factor. This hypothesis requires further study.

Independently, investigations of the long-term development of B cell malignancies in an another mouse model of GD, with GBA1 deficiency induced in hematopoietic cells (Gba^tm1Karl/tm1Karl^Tg(Mx1-cre)1Cgn/0). Established by mating Gba^tm1Karl/tm1Karl^ with Gba^tm1Karl/+^ Tg(Mx1-cre)1Cgn/0 mice [20]. These studies revealed sporadic fatal B cell lymphomas in 11 of 21 affected mice (6–24 months); in contrast, only two of eight control animals developed tumors by 24 months of age. Interestingly, most mice with overt lymphoma had absent or few Gaucher cells but local inflammatory macrophages were present. Eleven of the 39 GD mice developed monoclonal gammopathy, compared with only one animal of 25 in the control group. Seven of 10 with the B cell lymphomas were found to secrete a monoclonal paraprotein and the lymphomas stained intensely for pan-B cell markers. Reactive T lymphocytes were also present in tumor tissue. Subsequently, investigations were performed to examine abnormalities in glycoprotein non-Metastatic Melanoma B (gpNMB) in a GD mouse model with inducible knock down of glucocerebrosidase in the hematopoietic lineage by polyinosinic–polycytidylic acid treatment (Gba ^tm1Karl/tm1Karl^) [21]. The level of gpNMB was found to be markedly increased in plasma of these mice. [Investigators had previously demonstrated gpNMB is produced by Gaucher cells (lipid-laden macrophages), and that gpNMB levels correlated strongly with other established markers such as chitotriosidase and glucosylsphingosine.] The clinical significance of the increased plasma gpNMB found in patients with GD may involve the role of gpNMB in the degradation of cellular debris and macroautophagy [22]. Moreover, studies by Gabriel et al. revealed that lysosomal stress in a lipotoxic environment led to a substantial increase in gpNMB with induction seen at both the mRNA and protein levels [23].

## 4. Therapeutic Considerations

Systemic manifestations of Gaucher disease are currently managed using two approaches, namely, enzyme replacement therapy (ERT) and substrate synthesis inhibition (SSI; also known as substrate reduction therapy, SRT) [24]. ERT involves the regular intravenous infusion of a recombinant enzyme that is distinguished primarily from the endogenous enzyme, by having exposed mannose residues rather than mannose-6-phosphate residues (which facilitates cellular uptake of most of the other lysosomal hydrolases, such as α-galactosidase, which is deficient in Fabry disease, another disorder of glycosphingolipid metabolism). Earlier studies had shown α-mannosyl receptors are required for targeting the recombinant glucocerebrosidase to cells of monocyte/macrophage lineage, the primary cell type implicated in GD [25]. SSI entails the oral administration of a small molecule that partially inhibits the activity of ceramide-specific glucosyltransferase, thereby causing a reduction in the synthesis of the precursor for glucosylceramide (the substrate which is incompletely degraded in patients with GD and accumulates in cells of monocyte/macrophage lineage) [26].

Among GD patients on ERT, a statistically significant decrease in the percentage of polyclonal but not monoclonal gammopathies has been observed [14]. In mice, development of antibodies to injected human recombinant enzyme precludes studies of the therapeutic efficacy of ERT. Thus, examination of therapeutic outcome involving the mouse model has concentrated on evaluating the benefit derived from SSI therapy, primarily eliglustat. These experiments have indicated SSI therapy ameliorates the GD-associated gammopathy, likely achieved through a reduction in long-term immune activation by lysolipids. Treatment prevented the appearance of pathological macrophages and the proliferation of lymphocytes in the spleen and other organs. Importantly, late administration of the drug, that is, in GD mice after 7 months of age with established accumulation of glycosphingolipids tissues did not prevent lymphoproliferation, the appearance of monoclonal immunoglobulins, or the development of B-cell lymphoma [27]. The latter observation underscores the need to institute treatment in a timely fashion to maximize its benefits.

More recently, it has been reported SSI therapy given to the Gba ^tm1Karl/tm1Karl^ mice has been shown to result in a reduction of plasma gpNMB, correlated with the reduction of glucosylsphingosine [21]. Moreover, therapy with self-inactivating lentiviral vectors with the GBA gene (under the control of human phosphoglycerate kinase), which resulted in a functional correction of the GBA deficiency, promoted a reduction of gpNMB in liver, spleen, and bone marrow.

## 5. Summary

Gaucher disease, one of the more common lysosomal storage disorders, has been shown to be associated with an increased risk of malignancy, in particular, to monoclonal gammopathy and multiple myeloma. This propensity appears to be due to “chronic macrophage activation,” linked to the accumulation of incompletely metabolized substrates, glucosylceramide, and glucosylsphingosine, although additional studies require elucidation of mechanistic link(s). As the latter phenomenon occurs in all patients untreated, the factors that influence the expression of multiple myeloma in certain individuals are not understood. As this comorbidity is not encountered in children, a long period of exposure in “vulnerable” subjects may be at play. Enzyme replacement therapy has been shown to reverse hematopoietic and visceral organ manifestations of disease, although it has not eliminated the problem of gammopathy and evolution to multiple myeloma. Studies in mice models indicate that the risk of malignancy is prevented or reduced following treatment with substrate synthesis inhibition. Long-term follow-up of patients with GD treated with eliglustat will be required to ascertain whether similar benefits accrue.
